# Driving with Binocular Visual Field Loss? A Study on a Supervised On-Road Parcours with Simultaneous Eye and Head Tracking

**DOI:** 10.1371/journal.pone.0087470

**Published:** 2014-02-11

**Authors:** Enkelejda Kasneci, Katrin Sippel, Kathrin Aehling, Martin Heister, Wolfgang Rosenstiel, Ulrich Schiefer, Elena Papageorgiou

**Affiliations:** 1 Computer Engineering Department, Wilhelm-Schickard-Institute of Computer Science, University of Tübingen, Tübingen, Germany; 2 Centre for Ophthalmology, Institute for Ophthalmic Research, University of Tübingen, Tübingen, Germany; 3 Competence Centre “Vision Research”, Study Course “Ophthalmic Optics/Audiology”, University of Applied Sciences Aalen, Aalen, Germany; 4 Department of Ophthalmology, University of Leicester, Leicester Royal Infirmary, Leicester, United Kingdom; Harvard Medical School, United States of America

## Abstract

Post-chiasmal visual pathway lesions and glaucomatous optic neuropathy cause binocular visual field defects (VFDs) that may critically interfere with quality of life and driving licensure. The aims of this study were (i) to assess the on-road driving performance of patients suffering from binocular visual field loss using a dual-brake vehicle, and (ii) to investigate the related compensatory mechanisms. A driving instructor, blinded to the participants' diagnosis, rated the driving performance (passed/failed) of ten patients with homonymous visual field defects (HP), including four patients with right (HR) and six patients with left homonymous visual field defects (HL), ten glaucoma patients (GP), and twenty age and gender-related ophthalmologically healthy control subjects (C) during a 40-minute driving task on a pre-specified public on-road parcours. In order to investigate the subjects' visual exploration ability, eye movements were recorded by means of a mobile eye tracker. Two additional cameras were used to monitor the driving scene and record head and shoulder movements. Thus this study is novel as a quantitative assessment of eye movements and an additional evaluation of head and shoulder was performed. Six out of ten HP and four out of ten GP were rated as fit to drive by the driving instructor, despite their binocular visual field loss. Three out of 20 control subjects failed the on-road assessment. The *extent* of the visual field defect was of minor importance with regard to the driving performance. The *site* of the homonymous visual field defect (HVFD) critically interfered with the driving ability: all failed HP subjects suffered from left homonymous visual field loss (HL) due to right hemispheric lesions. Patients who failed the driving assessment had mainly difficulties with lane keeping and gap judgment ability. Patients who passed the test displayed different exploration patterns than those who failed. Patients who passed focused longer on the central area of the visual field than patients who failed the test. In addition, patients who passed the test performed more glances towards the area of their visual field defect. In conclusion, our findings support the hypothesis that the extent of visual field per se cannot predict driving fitness, because some patients with HVFDs and advanced glaucoma can compensate for their deficit by effective visual scanning. Head movements appeared to be superior to eye and shoulder movements in predicting the outcome of the driving test under the present study scenario.

## Introduction

Visual field defects usually occur with lesions involving the visual pathway. The pattern of visual field loss depends on the site of the lesion. (Advanced) glaucoma and post-chiasmal cerebral lesions are common disease entities associated with visual field loss in the binocular visual field. Glaucoma is the second leading cause of blindness in the Western world [1]. It is a characteristic optic neuropathy leading to (usually arcuate) visual field defects that follow the course of the affected retinal nerve fibers. In advanced stages of glaucoma the areas of monocular field defects may spatially coincide and thus result in binocular field loss. The central visual field (VF) and the visual acuity are usually spared even up to end-stage glaucoma. Post-chiasmal lesions of the visual pathway cause contralateral homonymous visual field defects (HVFDs), a loss of vision on the same side in both eyes, which respect the vertical midline. The most common causes for such HVFDs are stroke (70%), brain tumor and trauma [2].

### Glaucoma

Johnson and Keltner reported that persons with bilateral visual field defects (regardless of cause) had a higher frequency of motor vehicle collisions [3]. In an early study on the influence of glaucoma on driving ability, Owsley et al. investigated visual risk factors for vehicle crashes by elderly drivers [4]. The authors analyzed the number of accidents each participant was involved in during the last five years with respect to their age. They found out that the restricted useful field of view (UFOV) and glaucoma seem to be the only significant independent predictors of injurious crash involvement [4]. In 2004, McGwin et al. [5] reported that elderly people with glaucoma were safer on the road than their healthy counterparts due to the self-regulation of these patients (e.g., not driving by night or on rainy days). In a later work, the same authors [6] compared glaucoma patients who had a collision during an observation period of six years, to glaucoma patients who did not have a collision during the same period. Glaucoma patients with a collision were more likely to have moderate to severe visual field impairment. In 2008, Haymes et al. evaluated the on-road driving performance of glaucoma patients using a checklist completed by a driving instructor [7]. They found no significant difference between patients with glaucoma and control subjects [7].

### Homonymous visual field defects

Defects in the binocular visual field cause a marked amount of subjective inconvenience in everyday life. Patients with HVFDs may show persistent and severe impairments of reading, visual exploration and navigation, collide with people or objects on their blind side and may be deemed unsafe to drive. In Europe, Australia and over half of the states in the U.S., patients with HVFDs are not allowed to drive when they do not meet the minimum visual field requirements for licensure (e.g., 120° intact horizontal field along the horizontal meridian according to the European Community Directive on Vision and Driving, 2011). Since driving is of particular importance for maintenance of individual mobility, the driving ability of patients with binocular visual field loss has been investigated in several studies - both in simulated environments and on road. A variety of approaches has been used, such as self-assessment of subjects by means of questionnaires, evaluation of their driving performance by back-seated raters or analysis of scene camera recordings. However, the findings regarding the driving ability of patients with binocular visual field loss are controversial.

The driving ability of patients with HVFDs has been also investigated in several studies. Elgin et al. [8] investigated the driving safety of patients with homonymous visual field defects in six rating scales during an on road drive. They reported that although the drivers suffering from HVFDs received poorer ratings, they performed no or minor errors. Yet, a common problem for drivers with HVFDs was the lane keeping ability. Difficulties in keeping the lane position and gap judgment have also been reported in other simulator studies [9], [10]. Wood et al. [11] later developed a larger number of scoring criteria (e.g., lane keeping, steering steadiness, vehicle control, speed adjustment and reaction to unexpected events) that were evaluated by back-seat raters during the drive [11]. This approach was improved two years later by installing cameras on the roof of the car, in order to make the evaluation more objective and retrospectively replicable [12]. In addition, a camera was installed towards the face of the driver, in order to observe head, shoulder, and eye movements [12]. Their results showed that some of the patients with HVFDs, who were safe drivers, had similar visual field defects with patients who had failed the driving test. Thus, although some patients may suffer from identical visual field defects, their visual performance may vary and depend on their compensatory ability by eye and head movements. However, since no eye tracking has been performed in the above mentioned on-road studies, the evaluation of eye movements and compensatory strategies is lacking precision.

The aim of our study was twofold: (i) to assess the on-road driving performance of patients suffering from binocular visual field loss in comparison to age- and gender-matched healthy subjects by using a dual-brake vehicle, and (ii) to investigate the related compensatory mechanisms. This study is novel, because visual exploration was assessed by means of a mobile eye-tracker and several scene cameras recording both driver and scene. Thus, we were able to perform a quantitative analysis of eye, head, and shoulder movements.

## Materials and Methods

### Participants

Participants with binocular visual field loss were recruited from the Neuro-Ophthalmology Unit and from the Glaucoma outpatient care unit at the University of Tübingen (Germany). Normally-sighted control subjects were recruited from the Tübingen region and comprised group-age-matched volunteers.

To be included in the study, all participants were required to be at least 18 years old, have a Minimental Status Examination Score above 24, the ability to understand and comply with the requirements of the study, and normal function and morphology of the anterior visual pathways as evaluated by ophthalmological tests (fundus and slit-lamp examinations, ocular alignment, ocular motility). In addition, the age- and gender-matched control subjects should have normal visual fields, normal cup-to-disc ratio (less or equal to 0.5) and no history of brain injury or physical impairment. The best corrected monocular distant visual acuity of control subjects should be >20/20 for those aged-up to 60 years, >20/25 for those aged between 60–70 years and >20/33 for those aged more than 70 years. Patients' (HP and GP) best corrected monocular distant visual acuity should be at least 10/20. Time since lesion onset for HP should be at least six months. Exclusion criteria for HP were multiple sclerosis, Alzheimer's disease, Parkinson's disease, hemiparesis and visual hemi-neglect as determined by horizontal line bisection, copying of figures, and the “Bells test”. Glaucoma patients should suffer from open angle glaucoma with advanced binocular visual field loss (stages II–IV according to the Aulhorn classification [13]).

All participants were holding a valid driving license. After their stroke or accident many HP had quit or reduced driving to a minimum. However, all GP continued driving and only few of them had reduced the amount of driven kilometers per year during the last years.

The research study was approved by the Institutional Review Board of the University of Tübingen (Germany) and was performed according to the Declaration of Helsinki. Following verbal and written explanation of the experimental protocol all subjects gave their written consent, with the option of withdrawing from the study at any time.

Twenty eligible patients with binocular visual field defects, i.e., 10 with HVFDs (age 52.5±12.8 years) and 10 with glaucoma (age 60.7±8.7 years), and 20 normal-sighted control subjects (C), subdivided into the groups HVFD control subjects (HC, age 51±11.7 years), and glaucoma control subjects (GC, age 59.9±9.1 years) were finally enrolled into the study. In the HVFD group, there were four patients with right-sided HVFDs (HR) and six with left-sided HVFDs (HL).

### Vehicle and Route

**Figure 1:**
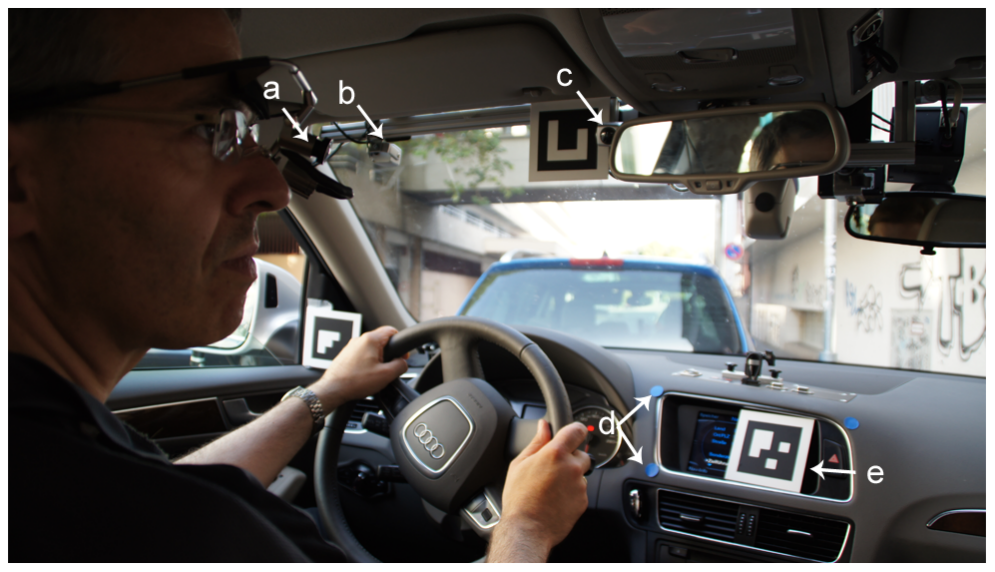
(a) A participant, wearing the mobile eye-tracking head unit; (b) First camera recording the road scene during the drive; (c) Second camera recording the driver during the drive; (d) Calibration points; (e) Scene marker used for calibration. The subject has given written informed consent, as outlined in the PLOS consent form, to publication of their photograph.

On-road driving performance was assessed under in-traffic conditions in an Audi Q5 SUV, provided by the Research Center for Computer Science (FZI) in Karlsruhe. The vehicle was equipped with a dual brake and two scene cameras. The first camera was directed to the road to record the forward driving scene Figure 1 (marked b) and the second was pointing directly towards the driver to record the pattern of head and shoulder movements Figure 1 (marked c). A certified driving instructor, who was aware of the medical status of the participants, sat in the front passenger seat and was responsible for monitoring safety. A second driving instructor, who was completely masked to the participants' medical and functional characteristics, sat in the right back seat and evaluated the driving performance.

The route was designed to meet the requirements of the German driving test procedure. The length of the course was 20 km including urban traffic, motorway, highways, and passages through residential areas, a traffic circle, some left-hand turn-off lanes, and backing into a parking space. Depending on the daytime and the traffic volume, each drive lasted between 30 and 40 minutes. Prior to the on-road assessment, the participants were introduced to the vehicle usage by the driving instructor and completed a short test ride.

Eight representative road scenes covering a wide range of traffic situations were selected. The chosen route sections with their particular challenges are listed in Table 1.

**Table 1:** Eight road scenes used for evaluation of driving fitness.

Scene	Name	Important factors
1	Urban traffic	respecting signs or signals, scanning, gap judgment
2	Merging into floating traffic	respecting signs or signals, exploratory head and shoulder movements, scanning, gap judgment
3	Roundabout traffic	scanning, gap judgment
4	Left turn	head and shoulder movements, gap judgment
5	Motorway	lane keeping, steering steadiness, speed
6	Highway access	speed, gap judgment, scanning, head and shoulder movements
7	Motorway	scanning, lane keeping, steering steadiness, speed
8	Backing into a parking space	gap judgment, speed, head and shoulder movements

### Eye Tracking

A mobile eye tracker (Dikablis, Ergoneers Inc., Manching, Germany) was used to record the eye movements during the driving task. The equipment is light-weighted and can be worn over the subjects' glasses. It consists of two cameras: one directed to the subject's eye to record the eye movements, and one directed to the road scene. Eye movements were recorded at a frequency of 25 frames per second. The head unit of the eye tracker was connected to a small transmitter mounted at the subject's neck. The transmitter coupled with the head unit had a wireless connection to the processing unit, which consisted of a receiver and the recording notebook. Due to its small and light-weight setup, the subjects were not hindered by the eye tracker during the on-road assessment.

During the experiment, two engineers ensured appropriate operation of the eye-tracker unit and the cameras by using the D-Lab Software (Ergoneers Inc, Manching, Germany) on a notebook. In addition to data recording and monitoring, D-Lab allowed for post-processing of the data (e.g., in case of insufficient pupil recognition) and for processing of the recordings.

Fixation clusters and saccades were recognized in the recorded eye data by means of an online learning algorithm based on Bayesian learning rules [14]–[16].

### Primary Parameter

The back-seated driving instructor (who was in the role of a driving examiner) assessed the driving performance of a subject as passed or failed according to the German driving license regulations. The test procedure is very strict, e.g., insufficient scanning, unstable position within the lane, inappropriate gap junction are already reasons for failure. Thus, a subject who manages to pass such a driving test is considered as safe driver. However, in case of a dangerous traffic situation or impending accident, where the driving instructor at the front seat took control of the vehicle, the driving test was immediately rated as failed. Each driving error was recorded by the back-seated driving instructor. The *Failure Rate*, (i.e., the percentage of drivers who failed the test), which was derived from the backseat instructor ratings, is the *primary* outcome criterion of this study.

### Gaze Parameters

In order to quantify the frequency and duration of saccades towards the visual field defect or towards the peripheral visual field, the visual field defect area of each patient was superimposed as an Area Of Interest (AOI). These models were transferred into D-Lab in order to analyze the viewing behavior of patients towards their “blind” visual field areas. D-Lab provides both overall and specialized analyses of gaze activity within a particular Area of interest (AOI). The following parameters were analyzed (Table 2):

**Table 2:** Studied parameters and their evaluation values.

Driving skills	Evaluation value
Lane keeping	from 0 = bad performance to 5 = very good performance
Speed	
Gap judgment (following distance, or distance to moving or parked cars)	
Scanning (Scanning and attention to other traffic participants or signs)	
Head movements	
Shoulder movements	from 0 = not excursive to 5 = highly excursive
**Gaze parameters**	
Eye movements	Horizontal gaze activity (HGA) in pixels
PGP in Areas Of Interest (AOI)	Proportion of Glances in Percent (PGP) towards AOI
Beyond the 30° visual field	
Beyond the 60° visual field	
Area of visual field defect	
Horizontal gaze distribution (HGD)	PGP and HGD values are average values of the eight evaluated scenes presented in Table 1.
L2: left peripheral area of the visual field (>60°)	
L1: left area of the visual field (20° to 60° left)	
C: central area of the visual field (20° left to 20° right)	
R1: right area of the visual field (20° to 60° right)	
R2: right peripheral area of the visual field (>60°)

**Figure 2:**
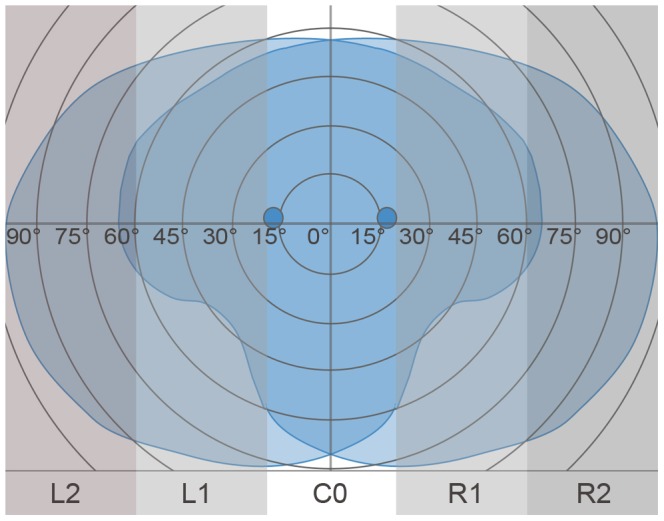
Distribution of the five different AOIs over the visual field for the assessment of HGD.

*Horizontal Gaze Activity (HGA):* Specifies the standard deviation of the pupil on the x-axis in pixel and was used as an overall measure of eye movements.
*Proportion of Glances in Percent (PGP):* The proportion of gazes towards a defined AOI during a specific time interval in percent.
*Horizontal Gaze Distribution (HGD):* The HGD describes the PGP in five different AOIs (Figure 2):
  - (L2) left peripheral area (>60°) of the visual field,
  - (L1) left area of the visual field between 60° and 20°
  - (C0) central visual field area between 20° left and 20° right
  - (R1) right area of the visual field between 20° and 60°
  - (R2) right peripheral area of the visual field (>60°) right.

### Driving Skills

The first two authors, who were aware of the drivers' visual field defect and their driving test results (passed/failed), conducted an analysis of the driving video material in order to assess further parameters such as lane keeping, speed, gap judgment, direction indicator usage, and overall scanning behavior. A 6-point rating system was used to assess the performance of the participants regarding these parameters (0 = bad performance, 5 = very good performance). In addition, the authors performed a quantitative scoring of head and shoulder movements on a 6-point scale (0 = not excursive, 5 = highly excursive). The percent agreement between both raters was K = 0.87. All evaluated parameters are listed in Table 2.

### Statistical Analysis

Statistical analysis was conducted using R [17]. In order to identify driving skills and gaze parameters associated with successful task performance, the above listed parameters were compared across the following participant groups: (i) control subjects who passed the driving test (C_p_), patients who passed (P_p_) and patients who failed the test (P_f_); (ii) glaucoma control subjects who passed (GC_p_), glaucoma patients who passed (GP_p_) and glaucoma patients who failed the test (GP_f_); (iii) HVFDs control subjects who passed (HC_p_), patients with right-sided HVFDs who passed (HR_p_), patients with left-sided HVFDs who passed (HL_p_) and patients with HVFDs who failed the test (HP_f_) by one-way ANOVA. All HP_f_ were patients with left-sided HVFDs. Subsequent post-hoc comparisons were performed using the Tukey's HSD test. As multiple tests were carried out, the significance level was adjusted using a Bonferroni correction to an alpha-level of 0.05 for multiple comparisons.

## Results

13 out of 40 (32.5%) participants failed the driving assessment: 4 out of 10 (40%) HP (0 HR and 4 out of 6 HL), 6 out of 10 (60%) GP and 3 out of 20 (15%) C.

The subjects, who failed the on-road assessment and the reasons for failures are listed in Table 3. Each subject is represented by an identifier, which is composed by the abbreviation of the participants' group (HL, HR, GP, C) and an unique identification number.

**Table 3:** List of participants who failed the on-road driving test and main reasons for failure.

*HL01*	Difficulties with gap judgment, lane keeping, speed and left turns
*C02*	Difficulties with lane keeping
*C12*	Speeding, difficulties with scanning, speed, and reaction to pedestrians
*HL15*	Difficulties with lane keeping and scanning
*HL27*	Difficulties with scanning, inadequate reaction to hazardous situations and difficulties following traffic lights
*C28*	Inadequate reaction at crossroads, difficulties with lane keeping, parking, and left turns
*HL33*	Difficulties with speed and scanning
*GP63*	Difficulties with gap selection, speed, scanning, turning, and inadequate reaction to dangerous situations
*GP65*	Difficulties following the speed limit
*GP67*	Difficulties following the speed limit, difficulties with lane keeping, scanning. Disregarded the right of way.
*GP73*	Intervention by the driving instructor, difficulties with scanning and lane keeping
*GP75*	Difficulties with lane keeping and following the traffic lights. Jumped a red light
*GP79*	Difficulties with scanning and following traffic lights

### Secondary Parameter Evaluation

**Figure 3:**
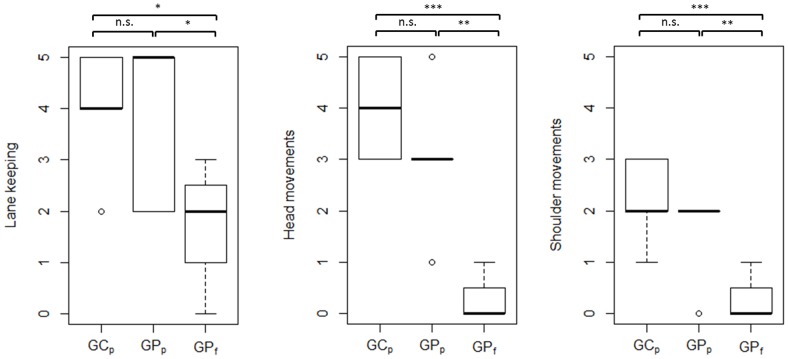
Driving parameters with significant differences between glaucoma control subjects who passed (GCp), glaucoma patients who passed (GPp) and glaucoma patients who failed (GPf) the driving assessment.

**Figure 4:**
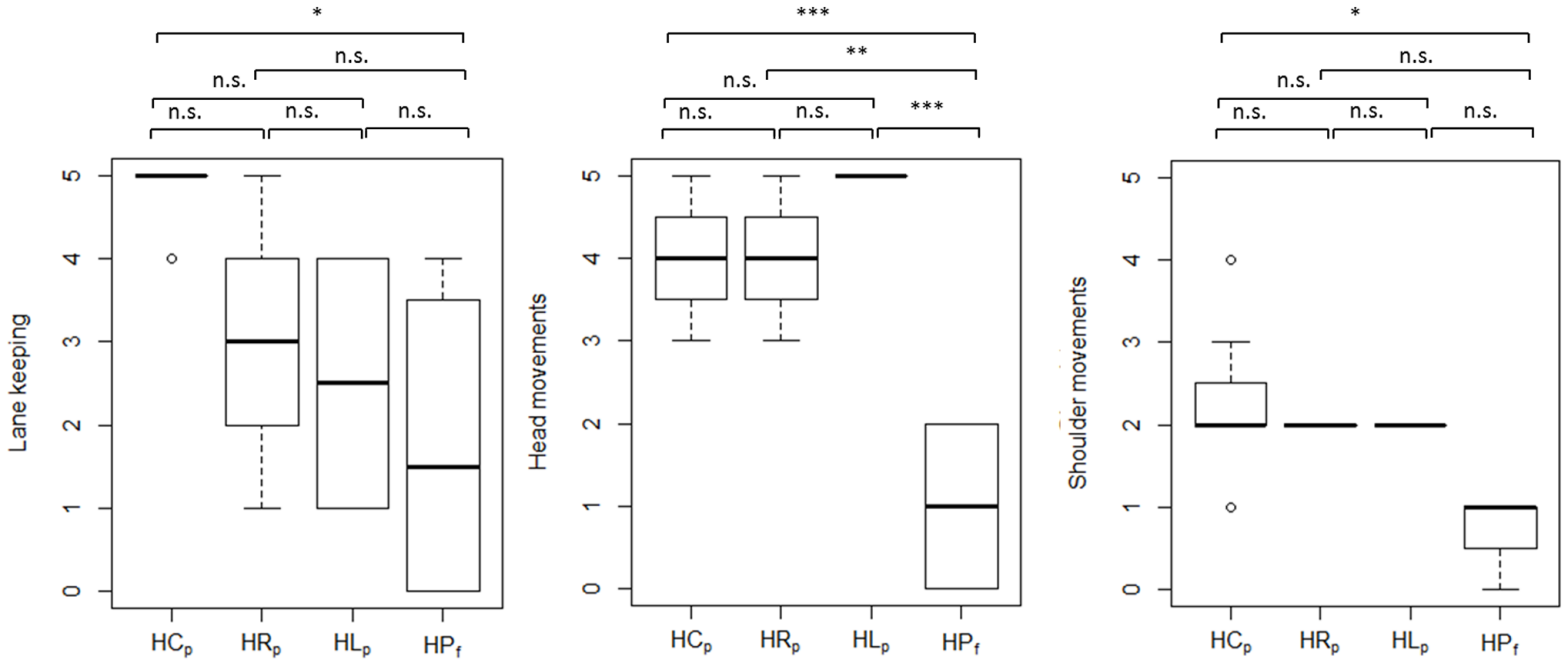
Driving parameters with significant differences between HVFDs control subjects who passed (HCp), patients with right-sided HVFDs who passed (HRp), left-sided HVFDs patients who passed (HLp), and HVFDs patients who failed (HPf) the driving assessment.

**Figure 5:**
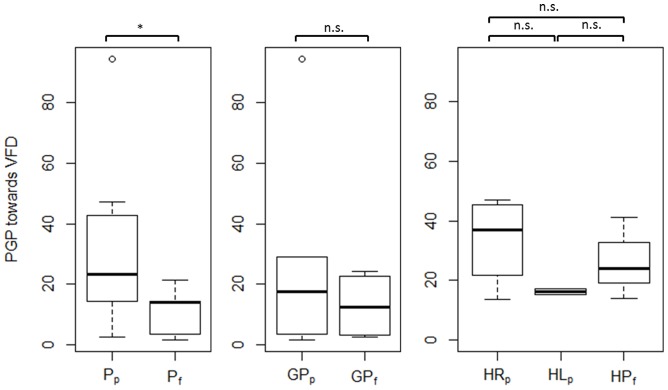
Left: Comparison of the proportion of glances in percent (PGP) towards the VFD between patients who passed (Pf) and patients who failed (Pf); Middle: Comparison of PGP towards VFD between glaucoma patients who passed (GPp) and glaucoma patients who failed (GPf); Right: Comparison of PGP towards VFD between patients with right-sided HVFDs who passed (HRp), patients with left-sided HVFDs who passed (HLp), and patients with HVFDs who failed (HPf).

**Figure 8:**
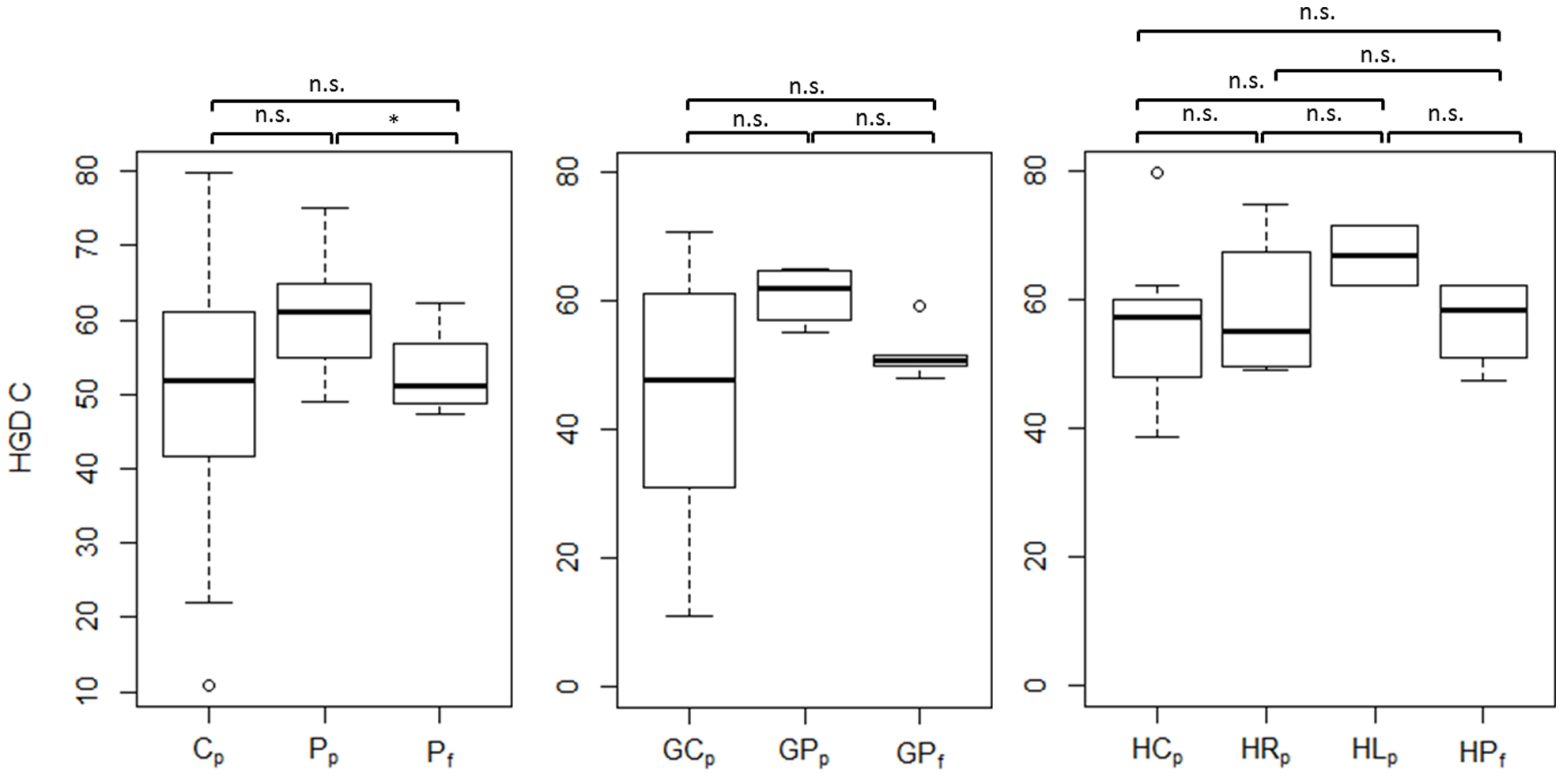
Comparison of the horizontal gaze distribution (HGD) towards the central 20° area of the visual field between patients who passed (Pf) and patients who failed (Pf); glaucoma patients who passed (GPp) and glaucoma patients who failed (GPf); and between patients with right-sided HVFDs who passed (HRp), with left-sided HVFDs who passed (HLp), and patients with HVFDs who failed (HPf).

Table 4 presents the results of the statistical analysis of driving skill and gaze-related parameters. Each parameter was compared across the following participant subgroups: (i) C_p_ – P_p_ – P_f_, (ii) GC_p_ - GP_p_ – GP_f_, and (iii) HC_p_ - HR_p_ - HL_p_ – HP_f_ by one-way ANOVA. However, since ANOVA does not explicitly reveal between which pairs of means there is a significant difference, the Tukey's HSD test was computed post-hoc. As multiple comparisons were carried out, a Bonferroni correction to an alpha-level of 0.05 was applied. Figure 3 and Figure 4 show between which pairs of subject subgroups significant differences in driving-related parameters were found. Furthermore, box plots were chosen to visualize each parameter. The bottom and top of each box represent the first and third quartiles, respectively. The band inside the box is the median. The whiskers represent the data within 1.5*IQR (Inter Quartile Range), whereas outliers are presented by single data points. Similarly, Figure 5 and Figure 8 show between which pairs of subject subgroups significant differences in gaze-related parameters were found. A detailed summary of the mean values of driving skill ratings, head, and shoulder movements, and gaze-related parameters is presented in Appendices 1 and 2 in Appendix S1.

**Table 4:** Driving skills and gaze-related parameters for control subjects who passed the driving assessment (C_p_), patients who passed (P_p_), patients who failed (P_f_), glaucoma control subjects who passed (GC_p_), glaucoma patients who passed (GP_p_), and glaucoma patients who failed (GP_f_), HVFDs control subject who passed (HC_p_), right-sided HVFDs patients who passed (HR_p_), left-sided HVFDs patients who passed (HL_p_) and HVFDs patients who failed (HP_f_).

	C_p_ - P_p_ - P_f_	GC_p_ - GP_p_ - GP_f_	HC_p_ - HR_p_ - HL_p_ - HP_f_
**Driving skills**			
Lane keeping	**	*	*
Speed	n.s.	n.s.	n.s.
Gap judgment	*	n.s.	n.s.
Scanning	*	n.s.	n.s.
Head movements	***	***	***
Shoulder movements	***	***	*
**Gaze parameters**			
HGA	n.s.	n.s.	n.s.
PGP beyond 30^0^	n.s.	n.s.	n.s.
PGP beyond 60°	n.s.	n.s.	n.s.
PGP towards VFD	*	n.s.	n.s.
HGD L2	n.s.	n.s.	n.s.
HGD L1	n.s.	n.s.	n.s.
HGD C	*	n.s.	n.s.
HGD R1	n.s.	n.s.	n.s.
HGD R2	n.s.	n.s.	n.s.

Statistical comparisons were made between groups.

* p<0.05,

** p<0.01,

*** p<0.001,

n.s. indicates non-significant comparisons. The significance level was adjusted using a Bonferroni correction to an alpha-level of 0.05 for multiple comparisons. HGA is the horizontal gaze activity in pixels. PGP describes the proportion of gaze in percent in a predefined area of interest (AOI), whereas HGD describes the horizontal gaze distribution in a predefined AOI (Table 2).

### Driving Skill Ratings

*Lane keeping:* Patients who failed the driving task (P_f_) performed significantly worse than control subjects who passed (C_p_) (p<0.01, **Table 4**). Lane keeping performance was significantly worse in GP_f_ than GP_p_ and GC_p_ (p<0.05, **Table 4**). Furthermore, HP_f_ performed significantly worse than HC_p_ (p<0.05, Table 4, Figure 4). This finding suggests that binocular visual field loss leads to difficulties in lane keeping.

*Speed:* All subject subgroups showed adequate performance regarding this parameter and no significant difference was found between patients' and controls' ratings.

*Gap judgment:* P_f_ showed significantly worse gap judgment abilities than C_p_ (p<0.05, Table 4). This result indicates that binocular visual field loss can lead to difficulties in maintaining a safe distance to moving or parked cars. Yet, there was no significant difference between the subgroups GC_p_ - GP_p_ - GP_f_ and HC_p_ - HR_p_ - HL_p_ - HP_f_.

*Scanning*: Regarding scanning and attention to other traffic participants or signs, patients who failed (P_f_) displayed significantly reduced scanning activity in comparison to controls who passed the test C_p_ (p<0.05, Table 4).

*Head and shoulder movements*: Subgroup analysis revealed that patients who failed the driving assessment (Pf) performed significantly less exploratory head and shoulder movements than control subjects C_p_ and patients who passed the driving test P_p_ (p<0.001, Table 4). Similarly, GC_p_ and GP_p_ performed significantly more head and shoulder movements than GP_f_ (p<0.001, Table 4, Figure 3). Subgroup analysis for HVFDs showed significantly more head movements in HC_p_ than HP_f_ (p<0.001, Table 4). HR_p_ and HL_p_ also displayed more head movements than HP_f_ (p<0.01, Figure 4). Regarding shoulder movements, HP_f_ performed significantly less shoulder movements than HC_p_ (p<0.05, Table 4).

### Gaze Parameters

*HGA (Horizontal Gaze Activity)*: Contrary to our expectations, no difference was found regarding the horizontal gaze activity between the participant subgroups.

*PGP (Proportion of Gazes in Percent)*: There was no significant difference between the subject subgroups regarding the proportion of gazes (PGP) beyond the 30° and 60° visual field. However, the percentage of glances towards the area of the visual field defect seems to interfere with the overall performance. Patients who passed the driving test (P_p_) performed significantly more glances towards their visual field defect than patients who failed (P_f_) (p<0.05, Table 4 and Figure 5).

**Figure 6:**
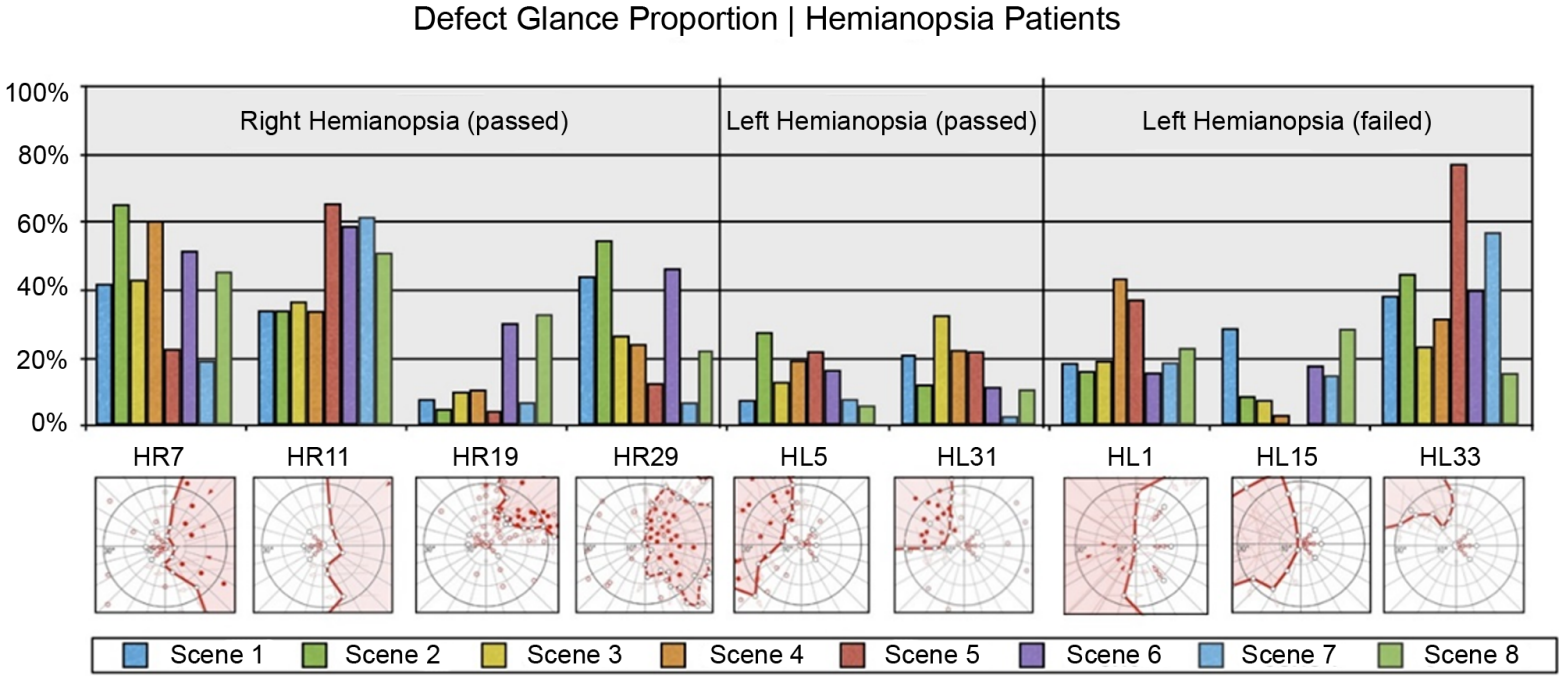
The proportion of glances in percent (PGP) of patients with homonymous visual field defects towards the VFD during the on-road assessment. For each of the eight road scenes (Table 1), the upper panel shows the PGP of each participant. The lower panel shows the corresponding VFD tested by binocular semi-automated 90° kinetic perimetry. The red line and the shallow red area represent the border and the extent of the HVFD, respectively.

**Figure 7:**
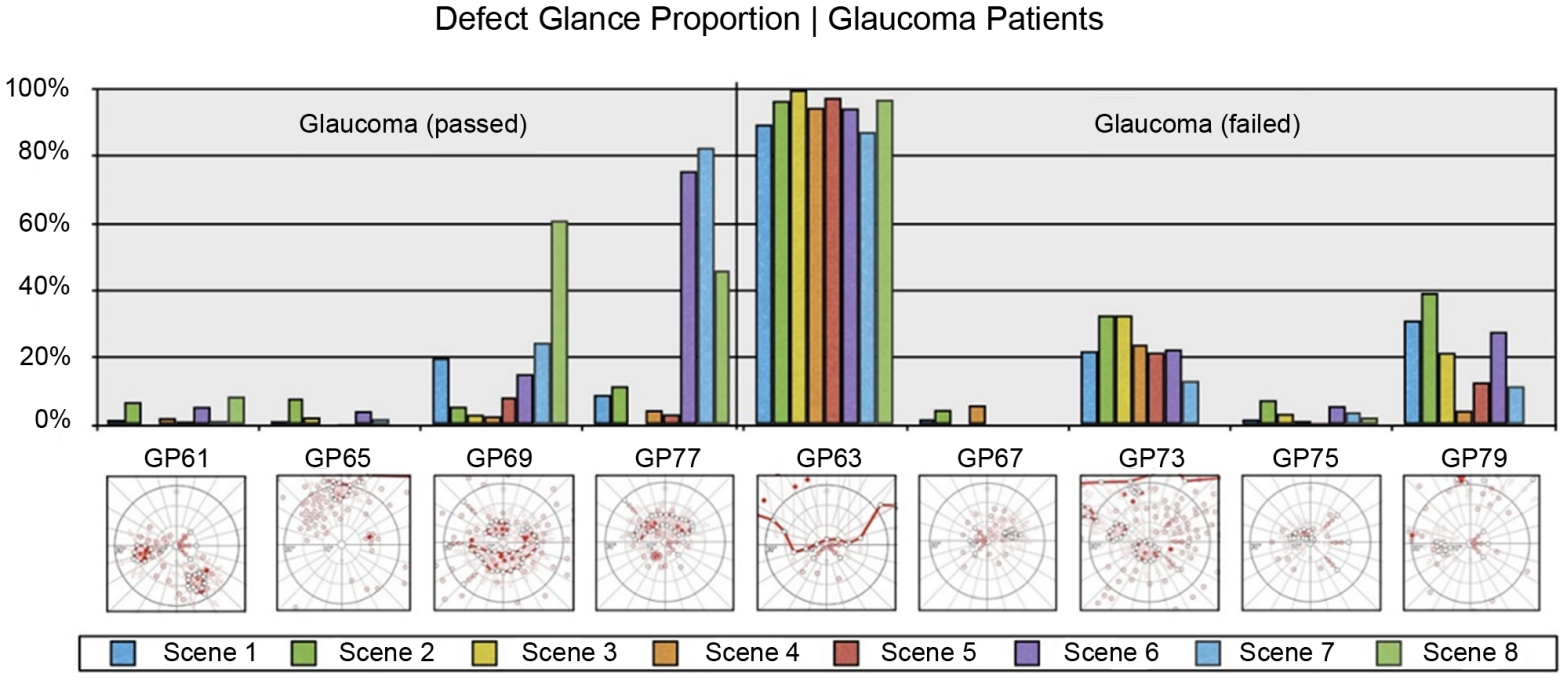
The proportion of glances in percent (PGP) of glaucoma patients towards the VFD during the on-road assessment (Table 2). Else, see Figure 6.

Figure 6 and Figure 7 present the proportion of gazes in percent (PGP) towards the visual field defect for HVFDs (HP) and glaucoma patients (GP), respectively. For HVFDs patients, the PGP towards the VFD is independent of the binocular VFD size (Pearson correlation HP: r = 0.5312, p = 0.1412, Figure 6). This is also applicable for the HL and HR subgroups (Pearson correlation HL: r = 0.6320, p = 0.2526; Pearson correlation HR: r = 0.8019, p = 0.1981). However, for glaucoma patients there was a significant correlation between the size of the VFD and the PGP towards it (Pearson correlation GP: r = 0.9611, p<0.001). Both Figures show that the size of the VFD cannot predict the driving performance of patients. However, due to the small number of participants in each subgroup, these results should be treated with caution and need further investigation.

*HGD (Horizontal Gaze Distribution):* There is no significant difference in the horizontal gaze distribution regarding the regions L2, L1, R1 and R2. However, patients who passed the test focused longer on the central area of the visual field than patients who failed the test, as indicated by HGD C (p<0.05, Table 4 and Figure 8).

**Figure 9:**
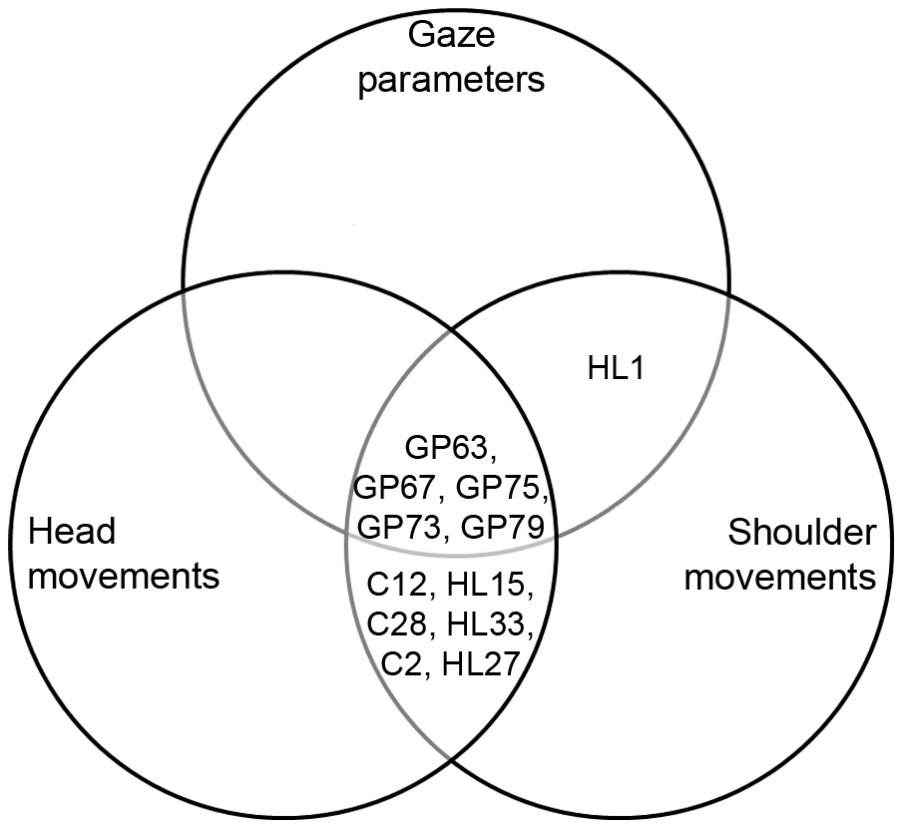
Venn diagram showing the distribution of subjects who failed the driving task among gaze parameters, head and shoulder movements regarding the capacity of these parameters in predicting failure.

**Figure 10:**
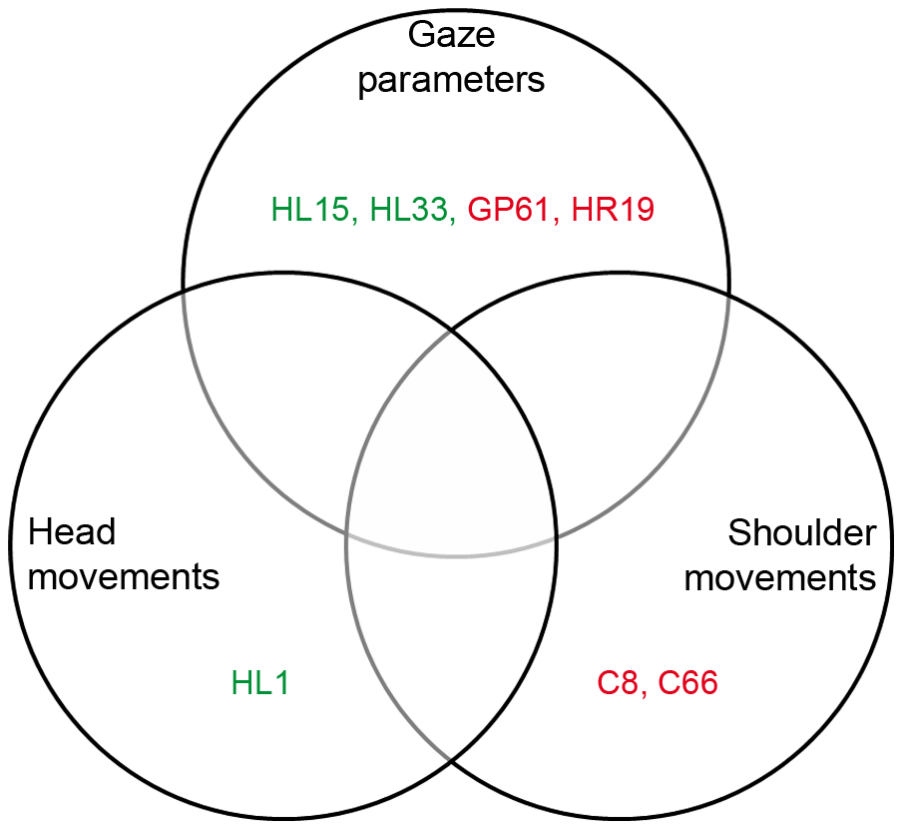
Venn diagram showing false-positives (red) and false-negatives (green) regarding failure prediction.

According to the gaze parameters, control subjects with IDs 2, 12, 28, and patients with left-sided HVFD with IDs 15, 27, 33 showed a good performance and were not rated as failed. However, head and shoulder movement analysis showed a poor performance of these subjects. One subject, namely HL1, showed good exploration ability regarding head movements, but poor performance regarding shoulder movements and exploratory eye movements. According to this evaluation, shoulder movements' assessment seems to be the best predictor of failure (Figure 9). However, considering the number of false-positives, i.e., subjects who were wrongly classified as failed, and number of false-negatives, i.e., subjects who were wrongly classified as passed (Figure 10), the best predictor for the outcome of the driving test under the present scenario is the assessment of head movements.

## Discussion

In this study we investigated the driving performance of patients with homonymous visual field defects and binocular glaucomatous visual field loss in comparison with age-and gender-matched normally-sighted control subjects, during an approximately 40-minute driving task on a pre-specified public on-road parcours. Our study is novel, because a quantitative assessment of eye movements by means of eye tracking equipment was performed.

A considerable number of patients managed to pass the driving test despite the binocular visual field loss. Six out of ten HVFDs patients and four out of ten glaucoma patients were rated as fit to drive by the driving instructor, despite not meeting the 120° horizontal visual field requirement. Interestingly, three out of 20 control subjects failed the on-road assessment. In two cases this was mainly due to speeding. One subject failed due to inappropriate parking distance and speeding on the left lane.

### Driving Performance and Effect of Visual Field Extent

#### Homonymous visual field defects

Previous studies have suggested that pass rates of drivers with HVFDs in on-road studies vary from 17% [18] to 73% [11]. Reasons for this wide variability have been the patient selection criteria, the experimental design and the specific driving situation tested. The majority of studies have highlighted poor steering control, incorrect lane position, difficulties in gap judgment and inadequate detection of potential hazards as the primary reasons for failing the driving tests [9]–[11], [19], [20], [23]. In addition, in a previous study investigating self-reported difficulty, drivers with hemianopic and quadrantanopic visual field loss expressed significantly more difficulties with driving maneuvers involving peripheral vision and independent mobility, compared to those with normal visual fields [21]. Our study is in accordance with the on-road study of Wood et al. [11], where 73% of patients with hemianopsia and 80% of patients with quadrantanopia were rated as safe drivers. The main problems in the above study were with lane position and steering control. Similarly, unsafe driving behavior in the present study was attributed to poor lane keeping and gap judgment ability. A recent study has indeed demonstrated that HVFD_S_ lead to impaired visual motor control specific to the axis of visual impairment [22]. In addition, we have demonstrated inadequate scanning behavior, defined as scanning and attention to other traffic participants or signs, as a main reason for failure of the driving assessment. Our study seems to be at odds with some on-road studies of drivers with HVFDs [20], [24], where less than 15% of patients passed the driving test. However, a patient selection bias may account for this difference, since these studies included patients who had been referred due to suspected driving safety concerns.

Furthermore, in agreement with previous on-road and simulator studies, we found that the *extent* of hemianopic visual field loss is of minor importance with regard to driving performance [11], [25]. Comparison of study participants with the largest visual field defects (ID01, ID07, ID11) indicates that the size of the visual field defect cannot be used as a decisive criterion for passing or failing the driving assessment. For example, ID01 has failed in the on-road assessment. However, ID07 and ID11 have passed the test, although the extent of their HVFD is similar. ID07 and ID11 suffer from right complete HVFDs due to a left occipital lobe lesion, whereas ID01 and ID27, who failed the task, demonstrate left complete HVFDs due to a lesion of the right occipital lobe.

All failed HP subjects suffered from left-sided homonymous visual field loss associated with right hemispheric lesions. In addition, patients with left-sided HVFDs had problems in traffic observation, resulting in difficulties with lane keeping, gap selection and speed, while patients with right-sided HVFDs showed mild deficits regarding the “left yields to right”-rule and lane keeping, especially during turning. Our results are in agreement with previous studies indicating that patients with right-hemispheric lesions perform worse on driving tasks, presumably because of a higher incidence of visuo-spatial deficits. The right hemisphere is assumed to be specialized for visuo-spatial function including visual search and the spatial guidance of eye movements [26]–[28]. In the present study, patients with clinical evidence of neglect or signs of impaired lateralized attention in the paper-and-pencil tests were excluded. However, it is possible that more subtle, subclinical attentional deficits were present in patients with right-sided lesions. More recent studies did not reveal significant differences in driving performance between patients with left- and right hemispheric lesions, and the small number of patients in this pilot study precludes further analyses [9], [11], [19], [25], [29]. Additional research is necessary in order to deliver more data about the influence of the lesion's side and location.

#### Glaucoma

Similar results regarding performance were also obtained for glaucoma, with four out of ten patients rated as safe to drive, despite not fulfilling the legal criteria for driving. Patients who failed the driving assessment had mainly problems with lane keeping, speed and scanning ability. There are only a few studies investigating the driving performance of glaucoma patients and a large body of literature has been based on self-reported accidents or police charts. In an early simulator study, Szlyk et al. found a higher incidence of real-world (self-reported) and simulator accidents among individuals with glaucoma compared to controls [30]. However, there were no differences in other measures of performance, perhaps because the authors used a general set of simulator performance indices and did not score performance for specific maneuvers. Our findings are in accordance with an on-road study of 28 drivers with glaucoma, where patients with worse VFs showed significantly poorer scores for changing lanes, driving around curves, and anticipatory skills [31]. In addition, our results are in partial agreement with a previous on-road study, where 60% of glaucoma patients – compared with 20% control subjects – had one or more at-fault critical interventions for reasons suggesting difficulty with detection of peripheral obstacles and hazards and reaction to unexpected events [7]. Apart from the present study, the study of Haymes et al. [7] is the only on-road study including a control group for glaucoma patients. Patients with glaucoma with slight to moderate visual field loss performed many real-world maneuvers safely. However, we found problems in important aspects of driving, mainly related to lane-keeping, speed and scanning. This difference may be attributed to the degree of visual field impairment, since the study of Haymes et al. only included patients who met the province visual standards for driving, having no more than moderate visual field impairment. In contrast, our patients had more severe glaucomatous visual field damage and did not meet the legal requirements for driving. In addition, the location of visual field defects may have played a role.

We have not found a strong association between the extent of glaucomatous visual field loss and fitness to drive, as shown in Figure 6. Not all studies investigating the association between glaucoma and traffic accidents have reported consistent results. In the on-road study of Bowers et al, the correlation between binocular visual field extent and overall driving performance was weak, while in the study of Haymes et al., worse eye MD in the glaucoma group was associated with the overall rating of driving. McGwin et al showed that older persons with glaucoma drive at least as safely as, if not more safely than, older persons without glaucoma [32]. In addition, McCloskey et al also found no evidence that glaucoma increases the risk of injurious collisions [33]. In addition, a simulator study reported that glaucoma patients with mild to moderate glaucomatous clinical vision changes did not have more accidents than the normally-sighted group [34]. The authors studied the relationship between visual field extent and driving performance failed to find a correlation between the binocular visual field and driving performance in a driving simulator [34].

### Gaze Patterns During Driving

#### Homonymous visual field defects

Therefore, our study adds to the increasing body of evidence that the extent of visual field per se cannot predict fitness to drive, because some patients are able to compensate by means of gaze scanning. Indeed we found that patients who passed the driving test (P_p_) showed a higher percentage of glances towards their visual field defect than patients who failed (P_f_). Those patients also demonstrated increased exploration in terms of head and shoulder movements and received superior ratings regarding scanning activity. Hence, our study confirms – by means of sophisticated eye-tracking – the hypothesis that effective scanning into the area of visual field defect is associated with superior driving performance. Accordingly, in an on-road study by Wood et al., patients with HVFDs rated as safe to drive also made more head movements into the blind hemifield and received superior ratings regarding eye movement extent [11]. Increased exploratory eye and head movements, particularly towards moving objects of interest on their blind side, were also evident in a simulator study assessing collision avoidance in patients with HVFDs [25]. Another recent descriptive simulator study suggested that patients with HVFDs who compensate for their visual impairment, perform an increased number of saccadic eye movements to the side of the VFD [35]. These authors did not identify head movements as part of the compensatory behavior in patients with HVFDs. However, this may be due to the more limited field of view used in this study.

A similar bias towards the side of the VFD in patients with HVFDs has been also observed in static visual search of images of everyday scenes. Patients with HVFDs shifted their gaze towards the side of the VFD, in order to bring a larger portion of the screen into the unaffected part of the visual field. This bias has been observed in several studies with static displays and may represent the attempt to compensate negative consequences of the visual field restriction with eye movements [20], [36]–[38].

Furthermore, patients rated as fit to drive, focused longer on the central area of the visual field than patients who failed the test, as indicated by HGD C. This interesting result is in agreement with a recent study, suggesting a significant bias of fixations and viewing time towards the center of the screen for both healthy subjects and patients with homonymous visual field defects during a visual search task in a static display. The authors suggested that this central bias could be related to functional specialization of the human visual field. Saccadic eye movements are performed in order to overcome acuity limitations of the visual field and shift its center to new objects of interest [41]. Several explanations have been suggested for the tendency to fixate the center of the scene when freely viewing images: First the central bias may result from motor biases that favor small amplitude saccades over large amplitude saccades. Second, the bias may arise from the distribution of image features. In addition, the center of the screen may be an optimal location for early information processing of the scene. Alternatively, the center of the screen may be a convenient location from which to start oculomotor exploration of the scene. Finally, the central bias may reflect a tendency to re-center the eye in its orbit [42].

#### Glaucoma

Gaze patterns of patients with glaucomatous visual field loss during driving tasks have been less studied. Patients with bilateral glaucomatous visual field loss made more saccades when viewing driving scenes in a hazard perception test, in an effort to compensate for their restricted field of view [39]. Another study by Cheong et al. examined traffic gap judgments and eye movements in subjects with peripheral visual field loss, including three subjects with glaucoma, as compared to healthy subjects [40]. They reported restricted gaze patterns but little evidence of difference in saccade amplitudes. In our subgroup of patients with glaucoma, no differences were found regarding the eye movement parameters between patients who passed and those who failed the driving assessment. However, this may be due to the small sample size, since PGP towards the visual field defect was significantly higher in patients who passed the test, when all patients (Glaucoma and HVFDs) were analyzed as a group. Interestingly, in glaucoma patients PGP towards the VFD was correlated to the size of the VFD, indicating an increasing percentage of glances towards the area of the visual field loss with more extensive visual field defects. Head and shoulder movements were also more pronounced in glaucoma patients who passed the driving test, highlighting the importance of active exploration in compensating for the VFD.

### Comparison of Eye vs. Head Movements in Predicting Fitness to Drive

Another interesting finding is the superiority of head movements over eye (gaze) movements in predicting the outcome of the driving test, which was observed in both glaucoma and hemianopic patients. Accordingly, in a recent on-road study patients with HVFDs rated as safe to drive made more head movements into the blind hemifield. These authors also conducted an analysis of the driving videos using a scoring system that allowed scoring of head movements. However, no quantitative eye movement analysis was available; therefore no comparison regarding the predictive value of eye vs. head movements was performed [12]. Our results are at odds with a previous study showing that hemianopic patients simplified search and fixation strategies by minimizing or entirely eliminating head movements and relying on eye movements instead. However, no driving task was performed in this study, and the target was presented in the horizontal plane within 10° of either side of center.

One might not expect a higher predictive value for head over eye movements, since the head is much larger in mass than the eyes, requiring more energy to move it [43]. In addition, head movements induce the vestibulo-ocular reflex (VOR). The semicircular canals measure head rotation velocity, and the signal they provide is fed to the eye muscles via the vestibular and oculomotor nuclei. The gain of this reflex is close to 1, so that a rotation of the head evokes an eye movement that almost exactly counteracts it [44]. However, the degree of eye-head coupling may depend on the task, as well as on experimental conditions [45]–[47]. Humans generally use a combination of eye and head movements to rapidly change the direction of the line of sight [46], [48]. A gaze shift of less than 15° is usually unaccompanied by a head movement in healthy volunteers, since additional energy is required to make head movements and stability of gaze is superior when the head is static [43]. The oculomotor range is approximately ±50°, meaning gaze shifts beyond this always necessitate a head movement to reach the visual target [43]. During large gaze shifts, the eye movement starts together with the head movement. The eye, however, reaches the target faster, and is subsequently stabilized by the VOR, while the head continues to move [49]. Driving is a challenging task which requires effective scanning of an extended area, especially in the horizontal plane. Therefore, a substantial contribution of head movements might be necessary in order to accomplish the task.

Indeed, examination of the eye and head movements of a racing driver (Tomas Scheckter) when driving at speed, revealed that his gaze was directed close to the tangent points of bends. Unlike low-speed driving this was almost entirely the result of head rotation, rather than eye-in-head movements, which were of low amplitude (<±10°) and almost unrelated to the head movements [50]. Overall, 92% of the gaze angle was brought about by changes in head direction, and only 8% was brought about by eye movements. It seems plausible, that the combination of eye and head movements depends in part on the size of the gaze rotation required. Patients with visual field defects, especially those with homonymous hemianopia, who additionally lack peripheral visual information that could guide saccades, need to perform even larger gaze shifts, in order to bring more visual information into the intact area of their visual field. This might lead to an increase of the relative contribution of head movements to gaze amplitude. Apart from the lack of peripheral visual stimuli in patients with visual field defects (bottom-up approach), another possible explanation is the presence of abnormal eye movements interfering with effective visual search in our subgroup of stroke patients (top-down approach). A previous study with four neurologic patients who showed hypometric saccades has also shown an increased relative contribution of the head to the total gaze shift, since the amplitude and velocity of the head movement were less affected [51].

These findings should be interpreted in the light of some study limitations. Despite the large total number of participants in our study (40 subjects), the number of participants in some of the subgroups was relatively small to derive significant differences between them (e.g., between left- and right-sided HVFD patients). For such subgroups, further studies involving a larger number of subjects would have to be conducted. Note that although HVFD and glaucoma patients are different regarding aetiology, they represent the two most common categories of patients who are denied a driving license. In light of the question whether a patient passed or failed a driving test, the outcome is more important than the underlying reason. A driving instructor is also unaware of the detailed medical history, when a patient undergoes a driving license test. We provided separate analysis for each group, however the results between the two groups of patients can be easily compared. For the detailed analysis of further eye movement parameters (e.g., saccade amplitude, frequency of smooth pursuits, etc.) mobile eye trackers at higher sampling rates would have to be employed. A better detection of the exact pupil position during driving, would lead to an improved scanpath analysis. In addition, driving skills were evaluated based on the video recordings and by post-test scoring by two independent researchers. Implementation of additional driving equipment could lead to more accurate vehicle recordings and quantitative inherent analysis of parameters such as steering steadiness, braking, and lane keeping.

## Conclusion

In conclusion, our findings support the hypothesis that the extent of visual field per se cannot predict fitness to drive, because some patients with HVFDs and binocular glaucomatous visual field loss can compensate for their deficit by scanning. Significant differences in lane keeping, gap judgments, and most importantly eye, head and shoulder movements were detected between patients who passed and those who failed the driving assessment. Patients who passed the test displayed different exploration patterns than those who failed, as they performed more glances towards their visual field defect and focused more on the central area of the visual field. In addition, they demonstrated more extensive head and shoulder movements. Head movements were superior to eye and shoulder movements in predicting the outcome of the driving test under the present study scenario.

The current challenge is to identify subjects who compensate for their visual deficit, by developing standardized realistic tools for individualized assessments in a clinical setting.

## Supporting Information

Appendix S1 **Appendices 1 and 2.**
(DOCX)
