# Supplementary material for: Driving with Binocular Visual Field Loss? A Study on a Supervised On-Road Parcours with Simultaneous Eye and Head Tracking
Source: PLoS One. 2014 Feb 11;9(2):e87470. doi: 10.1371/journal.pone.0087470 (PMC3921141; doi:10.1371/journal.pone.0087470)
Supplement: Appendix S1 — Appendices 1 and 2. (DOCX) [file pone.0087470.s001.docx]

# APPENDICES

Appendix 1 presents the mean values of driving skill ratings ( lane keeping, speed, gap judgment, blinker usage, scanning) and head and shoulder movements (0=bad performance to 5=very good performance).

| Parameter | passed  (Np=27) | failed  (Nf=13) | Control passed (n=17) | Control failed (n=3) | HR passed(n=4) | HL passed (n=2) | HL failed(n=4) | GP passed (n=4) | GP failed(n=6) |
| --- | --- | --- | --- | --- | --- | --- | --- | --- | --- |
| Lane keeping | 4.0 | 2.7 | 4.4 | 3.7 | 3.0 | 2.5 | 3.0 | 4.4 | 1.8 |
| Speed | 4.2 | 3.4 | 4.3 | 3.7 | 3.8 | 4.0 | 3.5 | 4.2 | 3.2 |
| Gap judgment | 4.8 | 3.9 | 5.0 | 4.7 | 4.3 | 5.0 | 4.5 | 4.4 | 3.0 |
| Direction Indicator | 4.9 | 4.7 | 4.9 | 5.0 | 4.8 | 5.0 | 4.5 | 4.8 | 4.6 |
| Scanning | 4.2 | 2.8 | 4.4 | 3.7 | 3.3 | 4.0 | 2.8 | 4.6 | 2.2 |
| Head movements | 4.0 | 1.0 | 4.0 | 1.7 | 4.0 | 5.0 | 1.3 | 3.5 | 0.4 |
| Shoulder movements | 2.1 | 0.6 | 2.1 | 1.0 | 2.0 | 2.0 | 0.8 | 2.0 | 0.2 |

Appendix 2 summarizes the mean values for gaze-related parameters: horizontal gaze activity (HGA), percentage glance proportion (PGP) towards several AOIs (60° visual field area, 30° visual field area, VFD) and horizontal gaze distribution (HGD) towards the areas L2, L1, C, R1 and R2 as described in Table 2.

|  | passed  (Np=27) | failed  (Nf=13) | Control passed (n=17) | Control failed (n=3) | HR passed(n=4) | HL passed (n=2) | HL failed(n=4) | GP passed (n=4) | GP failed(n=6) |
| --- | --- | --- | --- | --- | --- | --- | --- | --- | --- |
| HGA | 101,99 | 110,05 | 109,12 | 116,84 | 96,00 | 64,78 | 121,20 | 102,48 | 97,70 |
| PGP beyond 60° VF [%] | 10,99 | 8,47 | 12,81 | 9,85 | 8,11 | 4,29 | 7,88 | 10,36 | 8 |
| PGP beyond 30° VF [%] | 55,93 | 58,5 | 55,28 | 67,47 | 64,06 | 29,45 | 53,53 | 63,51 | 56,09 |
| PGP towards VF-defect [%] | 21,93 | 27,98 | - | - | 33,61 | 16,07 | 26,36 | 13,18 | 28,96 |
| HGD [%]  L2  L1  C  R1  R2 | 7,27 | 6,67 | 5,81 | 3,34 | 9,52 | 16,07 | 4,54 | 4,00 | 8,32 |
|  | 24,42 | 25,06 | 26,59 | 24,42 | 21,02 | 16,69 | 20,35 | 23,58 | 26,52 |
|  | 51,88 | 52,73 | 49,85 | 55,86 | 50,40 | 52,71 | 54,76 | 60,20 | 49,51 |
|  | 12,13 | 14,76 | 10,64 | 11,53 | 18,49 | 22,28 | 15,23 | 5,59 | 17,27 |
|  | 4,78 | 7,68 | 2,02 | 2,06 | 4,33 | 22,18 | 2,03 | 1,07 | 10,65 |
